# Supplementary material for: Impact of a Topical Anaesthesia Wound Management Formulation on Pain, Inflammation and Reduction of Secondary Infections after Tail Docking in Lambs
Source: Animals (Basel). 2020 Jul 24;10(8):1255. doi: 10.3390/ani10081255 (PMC7459688; doi:10.3390/ani10081255)
Supplement: Supplementary file 1 [file animals-10-01255-s001.pdf]

Table 1. Mean and SD of log^{10} cortisol prior to tail docking (C0), and at 30min (C1), 5hr (C2) and 48hr (C3) intervals post-tail excision, where "Surgery" was tail docking performed under general anaesthesia (Groups B&D), "Traditional" was tail docking conducted without general anaesthesia (Groups A&D), and 'yes' or 'no' was with or without Tri-Solfen®, treatment applied, respectively. Only significant differences were found for Groups A and C among time, being values for C1 significantly higher than the rest ($p<0.05$): ^{a,b} different letters mean significant differences for A, C Groups among time ($p<0.05$).

Time	Procedure	Pain Relief treatment (Group)	N	Mean±SD
C0	Traditional	No (Group A)	11	1.2±0.41 ^{a}
		Yes (Group C)	11	0.9±0.59 ^{a}
	Surgery	No (Group B)	11	0.9±0.38
		Yes (Group D)	11	1.0±0.51
C1	Traditional	No (Group A)	11	1.6±0.43 ^{b}
		Yes (Group C)	11	1.5±0.16 ^{b}
	Surgery	No (Group B)	11	0.8±0.46
		Yes (Group D)	11	0.8±0.50
C2	Traditional	No (Group A)	11	1.3±0.34 ^{a}
		Yes (Group C)	11	0.9±0.46 ^{a}
	Surgery	No (Group B)	11	1.0±0.53
		Yes (Group D)	11	1.0±0.51
C3	Traditional	No (Group A)	11	1.0±0.45 ^{a}
		Yes (Group C)	11	1.0±0.39 ^{a}
	Surgery	No (Group B)	11	1.0±0.42
		Yes (Group D)	11	0.9±0.59

Table 2. Mean and SD of log^{10} SAA at S0 prior to tail docking (S0), and at 5hr (S1) and 48hr (S2) intervals, plus seven days later (S3), where "Surgery" was tail docking performed under general anaesthesia (Groups B&D), "Traditional" was tail docking conducted without general anaesthesia (Groups A&D), and 'yes' or 'no' was with or without Tri-Solfen®, treatment applied, respectively. Only significant differences were found among time: ^{a,b}: different letters mean significant differences among time for traditional procedure ($p<0.05$); ^{A,B}: different letter mean significant differences among time for surgery procedure ($p<0.05$).

Time	Procedure	Pain Relief treatment (Group)	N	Mean±SD
S0	Traditional	No (Group A)	11	4.7±0.70 ^{a,b}
		Yes (Group C)	11	4.8±0.41 ^{a,b}
	Surgery	No (Group B)	11	4.2±0.87 ^{A}
		Yes (Group D)	11	4.2±0.69 ^{A}
S1	Traditional	No (Group A)	11	4.8±0.62 ^{a,b}
		Yes (Group C)	11	4.9±0.41 ^{a,b}
	Surgery	No (Group B)	11	4.4±0.89 ^{A}
		Yes (Group D)	11	4.3±0.41 ^{A}
S2	Traditional	No (Group A)	11	5.2±0.42 ^{b}

		Yes (Group C)	11	4.9±0.66 [b]
	Surgery	No (Group B)	11	5.0±0.39 [B]
		Yes (Group D)	11	5.0±0.46 [B]
S3	Traditional	No (Group A)	11	4.4±0.67 [a]
		Yes (Group C)	11	4.5±0.62 [a]
	Surgery	No (Group B)	11	4.6±0.40 [A]
		Yes (Group D)	11	4.3±0.54 [A]
